# Manic episode with psychotic symptoms in a patient with Pseudologia Fantastica of years of evolution. A case report

**DOI:** 10.1192/j.eurpsy.2024.1595

**Published:** 2024-08-27

**Authors:** C. Díaz Mayoral, E. Arroyo Sánchez, P. Setién Preciados, M. Martín de Argila Lorente

**Affiliations:** ^1^Psiquiatría, Hospital Universitario Príncipe de Asturias; ^2^Psiquiatría, Hospital Doctor Rodríguez Lafora, Madrid, Spain

## Abstract

**Introduction:**

Pseudologia Fantastica (PF) also called “mythomania” is a disorder centred on the tendency of the sufferer to distort reality through constant lies. These patients find it difficult to moderate their sense of self and their self-esteem. Therefore, they display significant grandiosity, which seems to defend them from intense psychological disturbance, pretending to counteract deep feelings of unworthiness, emptiness and alienation.

Notable characteristics include: normal or above average IQ, absence of formal thought disorder, poor sense of identity, poor sexual adjustment, low frustration tolerance, strong dependency needs and narcissism. The phenomenon of “imposture” (the person’s claim of achievement or having connections to famous or influential people) is frequent. The patient’s history often shows that one or both parents were experienced as rejecting figures. They are more likely to be involved in legal problems and 20% receive some form of psychiatric treatment.

The aetiology and pathogenesis of this disorder requires consideration of developmental disturbances, personal history and current life stressors.

**Objectives:**

A case of a patient with PF is presented followed by a theoretical review on the topic.

**Methods:**

A case is presented with a bibliographic review.

**Results:**

We admitted a 47-year-old man to the Acute Hospitalisation Unit for a suspected “psychotic episode with clinical mania”.

He presented manic and psychotic symptoms, with delusional ideation of months of evolution, megalomaniacal and fantastic discourse, centred on his work with high-ranking government officials and other implausible events. Multiple academic, work and personal life failures, with a diagnosis of depression 15 years earlier.

During admission, he constantly confirms his history. He tends to present a rationalising discourse and a minimising attitude towards behavioural alterations. He appears cooperative and docile at certain times, while at others he is irritable, complaining and threatening.

As for medication, olanzapine was initially prescribed at a dose of 20 mg per day, which was reduced to 10 mg given the psychopathological improvement and the difficulties of adherence.

On discharge, the presumptive diagnosis was “delusional disorder and probable personality disorder with narcissistic traits, with a history of PF, which in recent months has presented a manic episode with psychotic symptoms”.

**Conclusions:**

Their management poses challenges in terms of engaging with treatment and building a therapeutic alliance. It is important to assess the social and legal implications. Ensuring that they have stable relationships and adequate social supports is essential for successful treatment. Further exploration and research into this disorder is needed to better understand its manifestations and psychiatric consequences.

**Disclosure of Interest:**

None Declared

